# Balancing Work Life: Job Crafting, Work Engagement, and Workaholism in the Finnish Public Sector

**DOI:** 10.3389/fpsyg.2022.817008

**Published:** 2022-03-31

**Authors:** Terhi Susanna Nissinen, Erika Ilona Maksniemi, Sebastiaan Rothmann, Kirsti Maaria Lonka

**Affiliations:** ^1^Faculty of Educational Sciences, University of Helsinki, Helsinki, Finland; ^2^Optentia Research Focus Area, North-West University, Potchefstroom, South Africa

**Keywords:** job crafting, work engagement, workaholism, work balance, wellbeing, job resources-demands theory

## Abstract

The aim of this study was to investigate how job crafting, work engagement, and workaholism were related in public sector organizations. The participants (*N* = 213) were civil servants from three Finnish public organizations, representing different professions, such as school personnel, secretaries, directors, parking attendants, and ICT specialists. We duly operationalized job crafting, work engagement, and workaholism by using the Job Crafting Scale, the UWES-9, and the Work Addiction Risk Test. The current study focused on the Finnish public sector, since work engagement is recognized at the governmental level and has been shown to be strongly and positively associated with economic activity and productivity, while workaholism is associated with poor wellbeing. We analyzed the data by using structural equation modeling and found that three job crafting dimensions were strongly intertwined with one another. These dimensions were increasing structural job resources, increasing social job resources, and increasing challenging job demands. In the structural model, dimension “increasing structural job resources” was positively related to work engagement, whereas dimension “decreasing hindering job demands” was negatively associated with workaholism. This study highlighted the relevance of employees learning to balance their job resources and demands. We recommend that, in the public sector, employees be systematically encouraged to practice job crafting behavior by enabling them to increase structural job resources. These results are of high relevance, considering the heavy workload of public sector employees during the COVID-19 pandemic.

## Introduction

Public sector employees face work life demands in a work environment that is itself challenged by the need for high employee flexibility and widespread digitalization, as well as by having an aging labor force (Hazelzet et al., 2019). Therefore, the public sector institution must constantly evolve. However, the continuously changing public sector is different from the private sector. All municipalities are forced to look for new solutions in municipal services because of the changing needs of their residents and the lack of money. Employees’ resilience and competence, as well as their work contexts, set limits regarding possible solutions (Sotarauta et al., 2012). The present research concentrated on the public sector to explore job crafting strategies of employees that might be associated with work engagement and workaholism. Despite public sector importance, limited attention has been given to research on potential antecedents of work engagement and workaholism in public service organizations (Mostafa and Abed El-Motalib, 2020). Public sector contexts are rule-based, highly regulated, political, and contested. In addition, the public sector has high standards related to transparency and accountability, and the command chain can be complex (OECD, 2017). In order to deal with public sector features and demands to provide services successfully and to engage top professionals, there is a need to study balance in employees’ work lives by considering ways to craft job demands and resources. This is vital, especially after the long pandemic period and the extremely heavy workload among public sector employees.

To sustain a healthy work life in the public sector, it is essential to study how employees’ job crafting strategies in terms of job demands and resources affect their wellbeing (Le Blanc et al., 2017). Balancing is imperative from an organizational standpoint, as it reduces turnover and absenteeism caused by employee illness and increases productivity (Roczniewska et al., 2020). Inclusive and dynamic balancing will require mechanisms that can motivate employees, enhance their work engagement, and reduce negative outcomes, such as workaholism. Motivated by the complex public sector working life context, as well as theoretical and empirical contributions in earlier research, this study explored the role of job crafting in relation to work engagement and workaholism among public service employees.

Job crafting entails the changes that employees can make to improve their work (Wrześniewski and Dutton, 2001). Work engagement is defined as a work-intensive and long-lasting positive psychological state (Schaufeli et al., 2002, 2006; Schaufeli and Bakker, 2004, 2010), while workaholism indicates a strong, but compulsory, involvement in work (Ng et al., 2007; Upadyaya et al., 2016).

Research has shown that job crafting promotes work engagement, wellbeing, and organizational benefits and increases work performance, but it may also have a connection to workaholism (Wrześniewski and Dutton, 2001; Bakker and Demerouti, 2008; Schaufeli et al., 2008; Petrou et al., 2012; Tims et al., 2012; Kooij et al., 2015; Petrou et al., 2015; Bakker et al., 2016; Harju et al., 2016; Bakker and Albrecht, 2018; Kuijpers et al., 2020; Tóth-Király et al., 2020). Job crafting can strengthen competencies useful for career management and, consequently, moderate the level of job insecurity (Mazzetti et al., 2018) as well as create a pleasant work atmosphere (Tims et al., 2012) and enhance sustainable motivation (Tims and Bakker, 2010; Harju et al., 2016). Furthermore, job crafting often fulfills employees’ need for competence and relatedness (Bakker et al., 2016) and is useful in organizational changes. For example, Seppälä et al. (2020) showed that job crafting benefited work engagement among employees whose jobs had recently changed or whose work environment demanded changes. Job crafting may occur in professions of low or high autonomy (Wrześniewski and Dutton, 2001; Harju et al., 2016; Kuijpers et al., 2020), and organizations can stimulate job crafting behavior through human resource management (Bakker et al., 2016; Akkermans and Tims, 2017).

Even though there have been several previous studies on the topic, there is still a need to explore job crafting in relation to work engagement and workaholism because the biggest changes in 21st-century work have been in relation to the nature of work and the workforce (Kooij et al., 2015). In the present study, we explored job crafting as a tool for employees to adjust their work (e.g., high/low wellbeing and maintaining a sense of competence in relation to the demands of the work). We addressed job crafting dimensions (increasing structural job resources, increasing social job resources, decreasing hindering job demands, and increasing challenging job demands) as independent and combined variables in relation to work engagement and workaholism (Demerouti, 2014).

### Job Crafting

Job crafting is conceptualized from two dominant perspectives, namely, role-based crafting and resource-based crafting. Role-based crafting focuses on changes in work boundaries, work meaningfulness, and work identity (Wrześniewski and Dutton, 2001), whereas resource-based crafting focuses on job characteristics to balance job resources and demands in order to achieve good person–job fit (Tims et al., 2012). Both job crafting perspectives have demonstrated that employees can change aspects of their jobs to achieve person–job fit, higher work motivation, and well-being. Despite the different job crafting perspectives or terminologies (promotion- versus prevention-focused job crafting, or approach versus avoidance crafting), they describe employees enriching or reducing their job boundaries (Zhang and Parker, 2019.)

Job crafting can be operationalized through four dimensions, which are based on two types of job resources and job demands. Job resources are physical, mental, social, or organizational aspects of a job that typically reduce job demands or increase work-related goals (Wrześniewski and Dutton, 2001; Tims et al., 2012). Structural job resources are the range of resources that support work goals and stimulate personal growth and learning, such as work variety, opportunities for professional development, and autonomy at work (Bakker and Demerouti, 2007). Wessels et al. (2019) have also suggested the recognition of time-spatial resources, referring to employees actively selecting workplaces, work locations, and working hours. Social job resources are social activity, support, feedback, coaching or mentoring, and attaining interaction at work (Tims et al., 2012). Employees seek job resources the most when they experience considerable work autonomy and high work pressure (Petrou et al., 2012). A lack of these resources may lead to decreased work engagement and increased work stress (Hakanen et al., 2006).

Job demands are physical or mental efforts that employees experience in their job (Wrześniewski and Dutton, 2001; Tims et al., 2012), and they are associated with different outcomes, depending on whether they are hindering or challenging in nature (Crawford et al., 2010). Hindering job demands are physical or psychological efforts that are experienced as costs or overwhelming demands, such as emotional load or long working periods (Kooij et al., 2015). Challenging job demands are physical or psychological efforts that are rewarding and experienced as accomplishments (Kooij et al., 2015). Hindering demands, such as harmful organizational politics, may block professional progress, whereas challenging demands, such as new work projects, may promote professional progress (LePine et al., 2005). Challenging demands will encourage one to develop new competencies and increase challenging goals, but may also be experienced as a pressure (Demerouti et al., 2001; Bakker and Demerouti, 2007; Podsakoff et al., 2007; Crawford et al., 2010; Tims et al., 2012; Kooij et al., 2015; Bakker et al., 2016). Merely decreasing job demands is not a successful strategy for reducing exhaustion or increasing work engagement because decreasing such demands may affect burnout, but not work engagement (Petrou et al., 2012; Bakker et al., 2016; Van Wingerden et al., 2017b; Mäkikangas, 2018; Seppälä et al., 2020). Job demands may be stressful and challenging at the same time. This implies that lowering job demands may result in less challenging jobs and lower levels of work engagement (Petrou et al., 2015; Schaufeli, 2017b).

According to Hakanen et al. (2018), strategies to craft jobs by focusing on job resources and demands can be categorized as two types: expansive job crafting and decreasing job crafting (Wrześniewski and Dutton, 2001). Job crafting that is expansive involves a focus on increasing job demands (e.g., seeking challenges), a focus on finding structural resources, and seeking social resources. Decreasing job crafting pertains to actions intended to reduce hindering demands, such as avoiding colleagues who trigger stressful reactions. Tims et al. (2012) found that reducing hindering demands was unrelated to expansive job crafting strategies. Employees’ balancing between demands and resources and attempts to gain more resources are in line with the perspective of the conservation of resources (COR) theory (Hobfoll, 2001). According to the COR, people try to hold on to resources that are valuable to them, and individuals who accomplish good resources are in a better situation to invest resources, also in the future. The loss of resources is stressful because individuals have to face work demands with diminished coping capabilities (Hobfoll, 2001).

People who are engaged in expansive job crafting tend to experience their work more positively. They experience meaningfulness, have a better understanding of their work, make better decisions, and are more productive and efficient (Bakker and Demerouti, 2008; Bakker and Albrecht, 2018). According to the job crafting model (Tims and Bakker, 2010), expansive job crafting is associated with positive outcomes, such as work engagement (Tims et al., 2013; Hakanen et al., 2018). Reducing hindering demands is regarded as a mechanism to protect health when job demands are excessively high (Demerouti, 2014). Therefore, higher scores on the dimension “decreasing hindering job demands” can be expected to be associated with lower workaholism.

### Work Engagement and Workaholism

Employees who are engaged or suffer from workaholism are both heavy work investors (Salanova et al., 2014; Cheung et al., 2018). Previous research has drawn a motivational distinction between a positive form (work engagement) and a negative form (workaholism) of heavy work investment (Mazzetti et al., 2020).

Work engagement is a long-lasting positive psychological state that reflects wellbeing and work-related fulfillment, characterized by vigor, dedication, and absorption (Schaufeli and Bakker, 2004; Schaufeli et al., 2006; Seppälä et al., 2015). Vigor refers to high levels of energy, persistence, resilience, and willingness to invest in work (Tims et al., 2013). Dedication refers to enthusiasm, being strongly involved in work, and experiencing a sense of significance and challenge (Bakker, 2011). Absorption applies to the quality of deeply concentrating on and being focused on work (Tims et al., 2013). According to Bakker and Demerouti (2008), job resources and personal resources, independently or in combination, predict work engagement.

Like work engagement, workaholism also indicates strong involvement in work (Ng et al., 2007; Upadyaya et al., 2016). Strong involvement is associated with high job demands, which may lead to exhaustion, cynicism, and feelings of inefficacy (Demerouti et al., 2001; Hakanen et al., 2006; Crawford et al., 2010). Previous studies (Ng et al., 2007; Schaufeli et al., 2008; Choi, 2013; Molino et al., 2016; Upadyaya et al., 2016) have shown that the relationship between work engagement and workaholism is relatively strong; however, their outcomes are very different. High work involvement does not necessarily have negative effects or expose employees to the risk of workaholism if they are able to balance their job resources and job demands (Yu and Davis, 2016). Engaged employees work hard, and they differ from those who express symptoms of workaholism in the sense that they do not work compulsively, and they choose to do other things besides working in their spare time (Gorgievski et al., 2010).

Workaholism is regarded as the dark side of work engagement because the absorption dimension is often positively associated with workaholism (Schaufeli et al., 2008; Taris et al., 2010; Hakanen et al., 2012; Hakanen and Peeters, 2015; Clark et al., 2016). Thus, employees with strong work-related identities or who suffer from a lack of supervisory support or poor job control skills are at risk for developing workaholism (Schaufeli et al., 2008; Clark et al., 2016; Keller et al., 2016). Employees who experience high workaholism also suffer from physical and mental health problems, sleeping difficulties, work–family conflicts, burnout, and decreasing work performance and life satisfaction (Shimazu et al., 2015; Gillet et al., 2018). Instead, engaged employees frequently have a positive attitude, experience enthusiasm and good health, can shape their personal and job resources, and tend to spread their engagement to others, which will often, in turn, predict higher work performance (Bakker, 2011; Bakker and Sanz-Vergel, 2013; Bakker and Albrecht, 2018).

In previous research, work engagement was characterized as an optimal goal for both employer and employee (Bakker and Bal, 2010; Phelps, 2013). When employees gain more job resources, they experience high work engagement, and such circumstances enable job crafting (Bakker, 2011). A review by Knight et al. (2019) showed that bottom-up behavior, such as job crafting, was successful in promoting employees’ work engagement. Thus, active job crafting could be one prerequisite for stable work engagement (Hakanen et al., 2018).

Studies (e.g., Keller et al., 2016; Cheung et al., 2018) have shown that workaholism is related to heavy workloads, a competitive environment, and a lack of job resources. Therefore, decreasing hindering job demands can be expected to be negatively associated with workaholism. In addition, a study by Hakanen et al. (2018) showed that increasing structural job resources and challenging job demands were positively associated with workaholism. The latter findings can be explained as follows: individuals who suffer from workaholism invest a lot (even going beyond organizational expectations) in order to accomplish their tasks, start new projects, volunteer for additional tasks, and avoid situations that might prevent them from accomplishing their mission and goals (Hakanen et al., 2018). Therefore, people who suffer from workaholism feel that they need to perform better and constantly increase their structural job resources and challenging demands (Hakanen et al., 2018), which may prohibit them from restoring new resources. Also, they eventually become over-exhausted and withdraw from their work or decrease demands to protect and retain their resources (Schaufeli et al., 2009). This process will inevitably require even more investments in stress management and will prohibit workaholics from restoring gains (Hobfoll, 2001). To reduce the risk of slipping into workaholism, it is important to monitor one’s level of work engagement and, in that way, manage and maintain conditions for adjusting the level of engagement (Bakker et al., 2011).

### The Aims of the Study

According to earlier empirical and theoretical findings, job crafting, work engagement, and workaholism are significantly intertwined in work life. In this study, we explored their relationships further among public sector civil servants.

Based on earlier research, we expected to find positive associations between the job crafting dimensions, except for decreasing hindering job demands and the other three dimensions (Tims et al., 2012; Akkermans and Tims, 2017). We expected to find positive associations between expansive job crafting strategies and work engagement (Bakker, 2011; Tims et al., 2012; Bakker et al., 2016; Harju et al., 2016). Following longitudinal research of Hakanen et al. (2018) among Finnish dentists, we also expected to find positive associations between workaholism, on the one hand, and dimensions “increasing structural resources” and “challenging demands,” on the other hand. We assumed that employees who decreased their hindering job demands were strategic in their work balancing. Thus, we expected to find negative associations between dimension “decreasing hindering job demands” and workaholism. However, we did not expect to find associations between dimension “increasing social resources” and workaholism because previous research had shown deficiencies in workaholics’ social relationships (Hakanen et al., 2018). Therefore, the following hypotheses were set:

*Hypothesis 1 (H1)*: Different job crafting dimensions are positively associated with each other, except for decreasing hindering job demands.

*Hypothesis 2 (H2)*: Expansive job crafting strategies (increasing structural job resources, social job resources, and challenging job demands) and work engagement are positively associated.

*Hypothesis 3 (H3)*: Higher scores on the dimensions “increasing structural job resources” and “increasing challenging job demands” are positively associated with workaholism, and higher scores on the dimension of “decreasing hindering job demands” is associated with lower workaholism.

## Materials and Methods

### Context and Procedure

The present study was conducted in three public organizations in Finland, in both the governmental and municipal sectors. The questionnaire was language customized for each organization in collaboration with the organization’s contact person. During this customizing process, we paid attention to the terms and words that were used in the respective organizations to avoid common method bias. For example, we chose to use the word “team” instead of “group” if it was commonly used in the organization in question. By customizing the language and complementing this with clear instructions and a motivation letter, we made the questionnaire more face valid and relevant to the participants. The director in each organization recommended that the personnel answer the questionnaire, and participants were allowed to fill it out during their working hours. This recommendation indicated the importance of the study. For ethical reasons, participation was voluntary. With these procedural efforts, we tried to obtain accurate answers and increase the participants’ motivation to respond (Podsakoff et al., 2012). We conducted the research in an ethical and responsible manner, and it complied with all relevant legislation (Wager and Kleinert, 2011) of the European Code of Conduct for Research Integrity and the Responsible Conduct of Research (RCR) guidelines of the Finnish National Board on Research Integrity (TENK, 2019).

### Finnish Public Sector and Work Life

The Finnish public sector is known and valued for its efficiency, credibility, and corruption-free structures (Kaufmann et al., 2010). A success factor of the Finnish public sector is its personnel, but they have been challenged by insecurity and disruptions. The aim of the Finnish public sector is to develop and produce services in a sustainable and responsible way, so that the conditions for a good life can be secured not only for the present, but also for future generations.

The ongoing WORK2030 program is included in the Finnish governmental program, and its objectives for Finnish work life are to foster a work culture whose foundations lie in co-operation and trust, to make Finland a leading developer of work life innovations in the digital age, and to make Finland the world leader in wellbeing at work by 2030. In this continuous learning program, the Finnish government enhances workplace learning and networking and promotes competence development (Finnish Ministry of Social Affairs and Health, n.d.). These factors will affect the competitiveness of businesses and the effectiveness of public organizations (Schaufeli, 2017a).

### Participants

We approached 1,100 potential civil servants with this study and reached 213 voluntary participants from three public organizations. The response rate was 19.4%. Organizations A and B were in the field of education. The participants in Organization A were mainly highly educated teachers, teaching assistants, and administrative personnel from a special education school network. In Organization B, the participants were highly educated educational and administrative experts. Organization C was technical in nature, and the participants came from a wide variety of professional backgrounds, such as architects, park workers, parking supervisors, information and communications technology (ICT) experts, construction technology experts, administrative personnel, and customer service personnel. The total sample consisted of more females (39.9%) than males (19.7%), and the percentage of missing gender data was 40.4%. Only Organization C had more male participants than females (see Table 1).

**Table 1:** Descriptive statistics of the participants: *N*, gender, and years of work experience.

			Background factors		
Participants	*N*				
		Female, %	Male, %	Missing, %	Work experience, *M*
Total	213	39.9	19.7	40.4	12.0
Organization A	83	53.0	7.2	39.7	12.5
Organization B	38	50.0	21.1	28.9	12.6
Organization C	92	23.9	30.4	45.7	11.0

Missing = missing value for gender.

### Measures

#### Job Crafting

Tims et al. (2012) validated a four-factor job crafting scale (JCS), which was based on the job demands-resources (JD-R) model (Bakker and Demerouti, 2007). The JCS was modified in research by Petrou et al. (2017), Van Wingerden et al. (2017a), and Mäkikangas (2018). In this study, the first author translated the original 21-item questionnaire into Finnish and modified it for a Finnish context. We utilized this JCS-based scale to measure the four dimensions of job crafting by using a 19-item measure on a scale from 1 (*I totally disagree*) to 6 (*I totally agree*). We measured increasing structural job resources with four items in the questionnaire, such as “I make sure that I use my capacities to the fullest.” We combined the two original questions in this dimension, namely, “I try to develop my capabilities” and “I try to develop myself professionally,” into one item: “I try to develop my professional capabilities and my work.” Cronbach’s alpha was 0.83. We measured the decreasing hindering job demands dimension with six items in the questionnaire, such as “I make sure that my job is mentally less intense.” This dimension in the questionnaire included claims about mental, emotional, social, and cognitive behavior. Cronbach’s alpha was 0.66. The dimension increasing social job resources was measured with four items in the questionnaire, such as “I ask others for feedback on my job performance.” We omitted the original item “I look to my supervisor for inspiration” from this dimension. It included statements concerning general feedback on one’s work, asking one’s supervisor for guidance, asking colleagues for help, and asking one’s supervisor about satisfaction with one’s work results. Cronbach’s alpha was 0.66. The fourth dimension, increasing challenging job demands, had five items in the questionnaire, such as “If there are new developments, I am one of the first to learn about them and try them out.” Cronbach’s alpha for the fourth factor was 0.80. Tims et al. (2012) reported the following Cronbach’s alpha coefficients for the JCS: 0.82 for structural, 0.79 for hindering, 0.77 for social, and 0.75 for challenging.

#### Work Engagement

We used the Finnish version of the UWES-9 questionnaire (Schaufeli et al., 2006; Hakanen, 2009). The items were scored on a seven-point frequency scale from 1 (*never*) to 7 (*every day*). The UWES-9 measures three different dimensions of work engagement: vigor, dedication, and absorption. In the UWES-9, the internal consistency (Cronbach’s alpha) previously exceeded 0.85 (Schaufeli et al., 2006) and 0.95 in the Finnish version (Harju et al., 2016). In the present study, the internal consistency was 0.93.

#### Workaholism

We measured workaholism using the Work Addiction Risk Test questionnaire developed by Robinson (1999). The questionnaire included four items measuring excessive work and sense of duty. Items were scored on a scale from 1 to 7 as was the case with the work engagement scale. In the Finnish version by Upadyaya et al. (2016), the internal consistency (Cronbach’s alpha) exceeded 0.80. In the present study, Cronbach’s alpha for workaholism was 0.82.

### Data Analyses

For the statistical analyses, we used Mplus version 8.3 (Muthén and Muthén, 2018). Confirmatory factor analysis (CFA) was conducted to test the measurement model for the four job crafting dimensions presented by Tims et al. (2012). This was necessary because translation and modification altered the whole original scale. We also tested the full measurement model (which included job crafting, work engagement, and workaholism). To answer Hypotheses H1 to H3, we used a variable-centered approach, Pearson correlation coefficients, and a structural equation model (SEM) to explore associations between the four different dimensions of job crafting as well as their relations to work engagement and workaholism. In the SEM, we treated the four job crafting dimensions as independent factors. The work engagement and workaholism factors were treated as dependent variables. We assessed the internal consistency of the factors by computing the bootstrapped confidence intervals and point estimates of McDonald’s omega coefficients using the MBESS R package (Kelley, 2016). To assess model fit, we used the following fit indices: the chi-square/*df* ratio (*χ^2^*/*df*), the comparative fit index (CFI), the Tucker–Lewis index (TLI), the root mean square error of approximation (RMSEA), and the standardized root mean squared residual (SRMR). We utilized cutoffs of <0.06 for RMSEA, <0.08 for SRMR, and >0.90 for CFI and TLI (Hu and Bentler, 1999; Schreiber et al., 2006). Estimates were based on maximum likelihood with standard errors robust for non-normality (MLR), and we used full information maximum likelihood (FIML) to handle the missing data.

## Results

Table 2 shows the descriptive statistics and Pearson correlations for the four job crafting dimensions, work engagement, and workaholism. It shows that participants reported more work engagement (*M =* 5.75, SD = 1.11) than workaholism (*M =* 4.61, SD = 1.5) in the overall sample. Regarding the job crafting dimensions, participants reported increasing structural job resources the most (*M* = 4.55, SD = 1.20) and decreasing hindering job demands the least (*M* = 2.93, SD = 0.90).

**Table 2:** Descriptive statistics and correlations for job crafting, work engagement, and workaholism.

Variable	*N*	*M*	SD	1	2	3	4	5
1. Increasing structural job resources	201	4.55	1.20	–	–	–	–	–
2. Decreasing hindering job demands	201	2.93	0.90	−0.061	–	–	–	–
3. Increasing social job resources	200	3.33	0.95	0.381^**^	0.054	–	–	–
4. Increasing challenging job demands	201	4.03	1.21	0.659^**^	−0.109	0.385^**^	–	–
5. Work engagement	209	5.75	1.11	0.469^**^	−0.168^*^	0.239^**^	0.398^**^	–
6. Workaholism	208	4.61	1.50	0.183^**^	−0.240^**^	0.004	0.255^**^	0.210^**^

Six-point frequency scale in job crafting, seven-point frequency scale in work engagement, seven-point frequency scale in workaholism.

* *p* < 0.05;

** *p* < 0.01.

### Measurement Model for Job Crafting Dimensions

Our conceptual measurement model confirmed the four-factor structure of job crafting (see the model fits in Table 3). One 2-item cross-loading was allowed for the decreasing hindering job demands (Hindering) factor and another for the increasing social job resources (Social) factor. The first item pair, in the Hindering factor, was “I manage my work so that I try to minimize contact with people whose problems affect me emotionally” and “I organize my work so that I try to minimize contact with people whose expectations are unrealistic.” The second item pair, in the Social factor, was “I ask my supervisor to coach me” and “I ask others for feedback on my job performance.” These adjustments improved the model fit for the measurement model; this is referred to as the statistical model in Table 3. McDonald’s omega internal consistency for the scale increasing structural job resources (Structural) was 0.84, 0.76 for the scale decreasing hindering job demands, 0.68 for the scale increasing social job resources, and 0.84 for the scale increasing challenging job demands (Challenging). Internal consistency for work engagement was 0.96, and that for workaholism was 0.83. The scales (except for increasing social resources) had acceptable omega coefficients compared to the cutoff value of 0.70 (Nunnally and Bernstein, 1994).

**Table 3:** Summary of model fit.

	Fit indices										
	*χ* ^2^	scf	*df*	*p*	RMSEA [CI 95%]	CFI	TLI	SRMR	AIC	BIC	ABIC
*Job crafting*											
CFA conceptual model	293.029	1.1257	146	<0.001	0.071 [0.06, 0.08]	0.852	0.826	0.079	12600.944	12865.575	12665.981
CFA statistical model	226.557	1.1501	144	<0.001	0.053 [0.04, 0.07]	0.917	0.901	0.072	12592.159	12806.874	12600.944
*Job crafting, work engagement, and workaholism*											
CFA conceptual	733.729	1.0739	447	<0.001	0.055 [0.05, 0.06]	0.898	0.887	0.081	20698.709	21076.393	20718.349
CFA statistical	684.717	1.0699	446	<0.001	0.051 [0.04, 0.06]	0.915	0.906	0.080	20645.343	21026.369	20665.157
Structural model	684.717	1.0699	446	<0.001	0.051 [0.04, 0.06]	0.915	0.906	0.080	20645.343	21026.369	20665.157

scf, scaling correction factor for MLR estimator; AIC, Akaike Information Criterion; BIC, Bayesian Information Criterion; ABIC, sample-adjusted BIC. CFI, comparative fit index; TLI, Tucker-Lewis index; SRMR, standardized root mean square residual; RMSEA, root mean square error of approximation.

Concerning the correlations between the variables, Table 2 shows that dimension of “increasing structural job resources” was statistically significantly related to dimensions of “increasing social job resources” and “increasing challenging job demands,” work engagement, and workaholism. Dimension of “decreasing hindering job demands” was statistically significantly and negatively related to work engagement and workaholism. Furthermore, dimension of “increasing social job resources” was statistically significantly related to dimension of “increasing challenging job demands” and work engagement. Also, dimension of “increasing challenging job demands” was statistically significantly related to work engagement and workaholism, although the effect size of work engagement was larger. Finally, work engagement was statistically significantly related to workaholism.

### Associations Between the Four Job Crafting Factors

**Figure 1:**
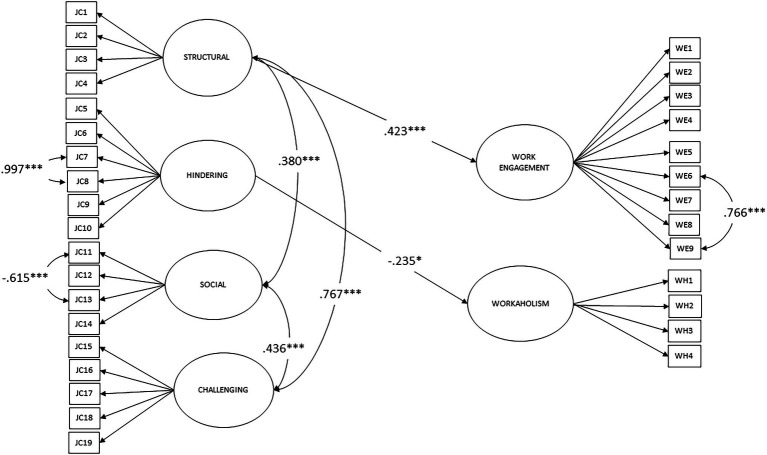
Structural equation model. Increasing structural job resources (Structural), decreasing hindering job demands (Hindering), increasing social job resources (Social), increasing challenging job demands (Challenging), Work Engagement, and Workaholism. ^*^p < 0.01. ^***^p < 0.001.

Figure 1 shows the results of the structural relationships between the latent constructs. According to the SEM analysis, the Structural factor was positively associated with the Social (*β* = 0.38, *p* < 0.001) and Challenging (*β* = 0.79, *p* < 0.001) factors. In addition, the Social factor was positively associated with the Challenging (*β* = 0.44, *p* < 0.001) factor. Associations between the Hindering factor and the other three factors of job crafting were not statistically significant. Thus, Hypothesis H1 was supported.

### Structural Model Linking Job Crafting, Work Engagement, and Workaholism

Good model fit was confirmed for the conceptual CFA model that linked the four job crafting dimensions, work engagement, and workaholism (see Table 3). One 2-item cross-loading was allowed for the Work Engagement factor: “When I am working, I forget everything else around me” and “I work intensely” (modification index = 44.07). Model fit for the structural model was acceptable (see Table 3). Figure 1 shows that, based on our model, the dimension of “increasing structural job resources” was positively associated with work engagement (*β* = 0.42, *p* < 0.001); thus, Hypothesis 2 was partly supported. Dimension of “decreasing hindering job demands,” in turn, was negatively associated with workaholism (*β* = −0.24, *p* < 0.05); thus, Hypothesis 3 was partly supported. Job crafting factors (the independent variables) predicted 31% of the variance in work engagement (*R*^2^ = 0.31, *p* < 0.001) and 12% of the variance in workaholism (*R*^2^ = 0.12, *p* < 0.05).

## Discussion

The present research explored job crafting in association with work engagement and workaholism among Finnish civil servants. Our study covered all job crafting dimensions, and increasing structural job resources was the most often reported dimension. Also, work engagement was reported more often that workaholism. These findings are in line with earlier meta-analysis (Rudolph et al., 2017), which had shown that increasing structural job resources explained more than half of the variability in work engagement. In our sample, we had more female participants, which might have affected the results in terms of job crafting, as women had previously reported higher levels of job crafting than men (Rudolph et al., 2017). It had also been shown that there was a positive relation between education and job crafting, except with decreasing hindering demands (Rudolph et al., 2017). We noted that Finnish citizens’ average educational background was relatively good due to the compulsory, free-of-charge education system. The sample in the current study was not highly educated overall. Consequently, our sample of Finnish civil servants might have affected the results. The Pearson correlation coefficients for all the variables are presented in Table 2, and Figure 1 shows the analyzed results of the structural relationships between all measured variables and latent constructs.

### Associations Between Job Crafting Dimensions

Our first hypothesis concerning associations between the three job crafting dimensions was supported. Table 2 and Figure 1 show that the three dimensions were positively associated with each other. It is possible that challenging job demands behavior encourages employees to develop in their jobs. Employees who seek more interesting and challenging goals in their career can show others that they are capable of extra challenges, and job crafting may play a mediating role between career competencies and career success (Akkermans and Tims, 2017). These kinds of mindsets and behaviors are related to increasing structural job resources, which support work goals, stimulate personal growth and learning, and provide variety or autonomy at work (Tims et al., 2012). According to previous studies, social resources are supervisor and colleague feedback, advice, or support and are often described as a response to employees’ need for relatedness in the work community (Pinto et al., 2014; Bakker et al., 2016). Work concerns relationships with other people, and these relations are part of the work identity process (Wrześniewski and Dutton, 2001).

Moreover, the results concerning the associations between the separate job crafting dimensions are in line with earlier findings that had shown that dimensions “challenging job demands” and “increasing job resources” were connected to social behavior (Akkermans and Tims, 2017). Our findings suggested that the fourth dimension, “decreasing hindering job demands,” was not related to learning (dimension of increasing structural job resources), professional development (dimension of increasing structural job resources), social activity (dimension of increasing social job resources), or new challenges at work (dimension of increasing challenging job demands). Decreasing hindering job demands may still be an important strategy when the challenge or work intensity is too high in relation to competencies (Inkinen et al., 2014) or when employees become overly engaged in their work. Premeditated decreasing job demands may help mature employees to work longer and stay healthy.

### Associations Between Job Crafting and Work Engagement

Our second hypothesis concerning associations between job crafting and work engagement was answered by Pearson correlation coefficients and by our SEM model. The coefficient findings supported Hypothesis 2 that expansive job crafting strategies were positively associated with work engagement. The present findings are in line with previous research showing that greater learning opportunities were the strongest predictor of work engagement (Sarti, 2014) and that employees were more engaged when they included increasing challenging job demands in their job crafting behavior (Bakker, 2011; Petrou et al., 2012; Harju et al., 2016; Bakker and Albrecht, 2018) or increased social job resources (Tims et al., 2012). In this study, we had a complex context and multidimensional variables, and when we estimated multiple and interrelated dependence between all variables through our SEM model, only one job crafting dimension had a significant positive association with work engagement, namely, “increasing structural job resources.” This finding might indicate that employees developed themselves professionally or modified the functional aspects and tasks of their work to achieve their work goals or to stimulate personal growth.

We found a negative association between dimension “decreasing hindering job demands” and work engagement. This finding is in line with an earlier study that had shown that a reduction in daily job demands had a negative association with daily work engagement and that reducing job demands might cause the job to become less motivating (Petrou et al., 2012). However, this association did not exist in intercorrelation relationships when we explored relationships between all measured variables and latent constructs. This latter finding might be due to the fact that decreasing burdensome job demands might remove some interesting aspects of one’s work and increase boredom (Petrou et al., 2012). The correlations in this study showed the association of dimension “increasing challenging demands” with work engagement (Demerouti and Peeters, 2018), although this association did not hold in the structural model. All findings considered appropriate balancing between demands and resources in work can promote work engagement.

### Associations Between Job Crafting and Workaholism

We found positive associations between dimension of “increasing structural job resources” and workaholism and between dimension of “increasing challenging job demands” and workaholism. This finding is in line with previous research that had found that workaholism was associated with increasing structural resources and challenging demands (Hakanen et al., 2018). With correlation coefficients, it is possible to point out the relationships between two variables, but as we stated earlier, the review of indirect relations is needed in studying multidimensional variables and a complex context. These positive association findings between dimension of “increasing structural job resources” and workaholism, as well as dimension of “increasing challenging job demands” and workaholism, were not found when we explored relationships between all measured variables and latent constructs in the structural model. We also found a negative association between dimension of “decreasing hindering job demands” and workaholism. Thus, Hypothesis 3 was partly supported. Our findings might imply a relationship between avoiding mentally intensive work or difficult decisions, which represent the dimension of “decreasing hindering job demands,” and low workaholism. Referring to earlier research, we could interpret the finding of dimension “decreasing hindering job demands” as presenting either work avoidance behavior (Mäkikangas and Schaufeli, 2021; Petrou and Xanthopoulou, 2021) or constructive optimizing behavior to make work more efficient (Demerouti and Peeters, 2018). However, findings regarding emotionally demanding jobs reported more avoidance of the strenuous aspects of the job (minimizing) than attempts to make work more efficient (optimizing; Demerouti and Peeters, 2018). Our study explored associations between job crafting behaviors and workaholism, and the results suggested that decreasing hindering job demands decreased workaholism. We suggest that decreasing hindering job demands is worth noting as one valuable strategy for optimizing job demands and resources.

### Limitations

The first limitation of this study concerns the small sample size (*N* = 213) and low response rate, type of employees (Finnish civil servants), gender distribution (the majority being women), and a considerably high percentage of missing values in gender (40%), which was due to the participants’ concern about their anonymity. More studies would be needed in future to explore similar associations in larger data sets.

Second, participation in the study was voluntary without any inducements, which might have resulted in missing values and a sample bias toward employees who were more engaged in their work. Thus, it was not possible to generalize our findings to all Finnish civil servants due to the limited population. However, we made an effort to prevent sample bias by sampling from more than one organization and managed to strengthen our data with some variety in occupations. Our findings are also in line with several previous research studies rooted in the same theoretical background. Moreover, it is possible that there are homogeneous subgroups of employees using different job crafting strategies (Mäkikangas, 2018). Future research could adopt a person-oriented approach to examine job crafting profiles in relation to wellbeing and work performance and in different nationality groups.

Third, only self-report measures were used in the present study. This might give rise to the question whether the participants behaved according to their answers, and therefore, common method bias was possible (Podsakoff et al., 2003; Conway and Lance, 2010). We did, however, consider widely used, reliable, and valid self-report questionnaires to be justified because these variables reflected the subjective experiences of the participants. These variables could have been difficult for others (supervisor, peers, researcher) to measure or time-consuming to measure through other methods (Wrześniewski and Dutton, 2001).

Fourth, the data were cross-sectional, preventing us from drawing conclusions about how our findings might change or progress over time. The correlations and structural equation model findings obviously indicated nothing about causality. The slightly lower alpha values of two factors (decreasing hindering job demands and increasing social job resources) might have occurred because these dimensions consisted of items measuring work avoidance and social crafting in many different areas. The low value of internal consistency can be seen as a limitation of this study. Using a longitudinal design in our future research would allow us to observe some temporal trends regarding how job crafting predicts work engagement or workaholism and whether difficult circumstances, such as the COVID-19 pandemic, change job crafting in the public sector.

### Practical and Theoretical Implications

The present study has various implications for job crafting theory and for public sector employers and employees. Most job crafting studies have focused on work engagement as an outcome of job crafting, and only a few studies have examined job crafting and workaholism. Due to our finding of a negative relation between dimension of “decreasing hindering job demands” and workaholism, we suggest that decreasing hindering job demands is noted in theory and in practice as one possible strategy for optimizing job demands and resources. The present findings showed that dimension of “increasing structural job resources” and work engagement were positively associated. According to this finding, we suggest enabling and managing especially structural job crafting among civil servants to affect their work engagement. However, merely encouraging employees to try out new things at work may not increase job crafting (Wolfson et al., 2018), and employees should also be involved in systematic training (Junell and Ståhle, 2011). In addition to creating wellbeing strategies and stimulating individual job crafting, we recommend job crafting intervention programs as an organization-level action (Van Wingerden et al., 2017a). Adjusting job demands and resources is vital for retaining employees’ ability to continue working (Le Blanc et al., 2017), especially in unprecedented conditions, such as during the COVID-19 pandemic that has drastically increased the workload.

## Data Availability Statement

The raw data supporting the conclusions of this article will be made available by the authors, without undue reservation.

## Ethics Statement

Ethical review and approval was not required for the study on human participants in accordance with the local legislation and institutional requirements. The participants provided their written informed consent to participate in this study.

## Author Contributions

TN conceived the research project, conducted the data collection, reported the results, interpreted the findings, and wrote the manuscript. SR provided guidance on conducting the SEM analysis that TN and EM performed together. SR and KL revised the manuscript. KL guided and oversaw the research. All authors contributed to the article and approved the submitted version.

## Funding

We are grateful for projects funded by the Academy of Finland (308352) and the Finnish Strategic Research Council (327242) in terms of the project infrastructure.

## Conflict of Interest

The authors declare that the research was conducted in the absence of any commercial or financial relationships that could be construed as a potential conflict of interest.

## Publisher’s Note

All claims expressed in this article are solely those of the authors and do not necessarily represent those of their affiliated organizations, or those of the publisher, the editors and the reviewers. Any product that may be evaluated in this article, or claim that may be made by its manufacturer, is not guaranteed or endorsed by the publisher.
